# Detection of *Plasmodium* spp. in Human Feces

**DOI:** 10.3201/eid1804.110984

**Published:** 2012-04

**Authors:** Milan Jirků, Kateřina Pomajbíková, Klára J. Petrželková, Zuzana Hůzová, David Modrý, Julius Lukeš

**Affiliations:** Author affiliations: Biology Centre, Institute of Parasitology, ASCR, České Budějovice, Czech Republic (M. Jirků, D. Modrý, J. Lukeš);; Faculty of Science, University of South Bohemia, České Budějovice (M. Jirků, J. Lukeš);; University of Veterinary and Pharmaceutical Sciences, Brno, Czech Republic (K. Pomajbíková, D. Modrý); CEITEC, Brno (D. Modrý);; Institute of Vertebrate Biology, ASCR, Brno, and Zoo Liberec, Liberec, Czech Republic (K.J. Petrželková);; Health Institute, Prague, Czech Republic (Z. Hůzová)

**Keywords:** Plasmodium spp., malaria, great apes, diagnosis, human feces, parasites

## Abstract

Comparison of diagnostic methods for *Plasmodium* spp. in humans from Uganda and the Central African Republic showed that parasites can be efficiently detected by PCR in fecal samples. These results, which rely solely on PCR-based examination of feces, validate numerous estimates of the prevalence of malaria in great apes.

In spite of a century of research, knowledge of *Plasmodium* spp. affecting the African great apes is limited. However, molecular tools have recently shown unexpectedly high diversity of these species (*1**–**5*). Available evidence supports the scenario in which humans acquired *P. falciparum* from western gorillas (*3**,**6*) and carried it in their blood throughout the world after migrating from Africa (*7*). However, recent discovery of a *P. falciparum*–related parasite in the African putty-nosed monkey calls into question this theory of the origin of this most malignant and widespread *Plasmodium* species (*8*). All data for identification of *Plasmodium* spp. were obtained only by PCR-based amplification of feces of free-living great apes (*9*). Because it is difficult to obtain their blood samples, which are used from for detection of *Plasmodium* spp. in other hosts, estimation of the prevalence of *Plasmodium* spp. in great apes is problematic.

*Plasmodium* spp. cause >200 million malaria cases in humans (*10*). The availability, sensitivity, and accuracy of diagnostic methods often rely on use of blood samples (*11*), making any attempts to inspect other material from humans for these pathogens unnecessary or impractical. Furthermore, a blood parasite would not be expected to be present in feces at detectable amounts (*12*). No attempts to identify *Plasmodium* spp. in feces of infected persons have been reported.

Studies of great apes (*3**,**4**,**9*) have focused on diversity and phylogeny of *Plasmodium* spp. However, fecal-based diagnostics open a plethora of questions regarding the prevalence, epidemiology, and clinical role of malaria in apes. To answer these questions, we urgently need to assess the reliability of fecal-based diagnostics. Motivated by prior feasibility of such an approach in closely related great apes, we examined whether malarial infection is detectable in feces of infected humans, and if so, in what fraction of infected persons does the parasite penetrate into feces?

## The Study

We analyzed fecal samples from 16 patients given a diagnosis of malaria at Lwanga Hospital in Buikwe, Uganda, who were undergoing treatment during October–December 2010; twenty-eight employees of Dzanga-Sangha Protected Areas, Central African Republic, who when samples were obtained during November–December 2010, did not show any symptoms of malaria but were considered a high risk group; and 6 Europeans who had repeated cases of malaria during 2003–2010 (samples were provided in April 2011). Samples obtained from 2 Europeans who never visited regions to which malaria was endemic were used as negative controls. Blood and fecal samples were obtained from each person. Blood smears were also prepared from the 16 patients in Uganda. We adhered to the research protocol defined by Dzanga-Sangha Protected Areas. Permission to collect samples was obtained from all examined persons before samples were obtained.

Thick blood smears were prepared by spreading 2–3 drops of fresh blood on a slide. Slides were dried, stained for 20 min with a 1:9 dilution of Giemsa, washed with tap water for 3–5 min, dried, and inspected for *Plasmodium* stages by light microscopy (100 fields at a magnification of ×1,000). Parasitemia levels in positive samples were determined according to standard protocols (*13*). Feces and blood were fixed in 96% ethanol and stored at room temperature until processed by using the QIAamp DNA Stool Mini Kit (QIAGEN, Hilden, Germany). DNA from blood samples stored on filter paper in ethanol was isolated as follows. The ethanol was evaporated; the filter paper was then transferred into 200 μL of 5% Chelex 100 (Sigma, St. Louis, MO, USA), incubated at 56°C for 1 hour, boiled for 10 min, and stored at 4°C. Before use, samples were centrifuged for 1 min at 15,000 rpm.

We amplified part of the apocytochrome (*cyB*) gene of *Plasmodium* spp. by using a reported protocol (*9*) and primers DW2-F and DW4-R in a first-round PCR and primers CYTB1-F and CYTB2-R in a second-round nested PCR. The amplified 938-bp fragment was resolved by agarose gel electrophoresis (Figure). Samples were subsequently blotted onto membranes and hybridized with the same PCR product labeled by random priming with ^32^P-dATP (ICN, Costa Mesa, CA, USA). The membrane was hybridized at 65°C overnight and washed 3× in SSC (0.15 M NaCl, 0.015 M sodium citrate, 0.1% sodium dodecyl sulfate) at 65°C (20 min/wash), and analyzed by using a phosphoimager. The *cyB* gene of *P. falciparum* was used as a positive control.

**Figure:**
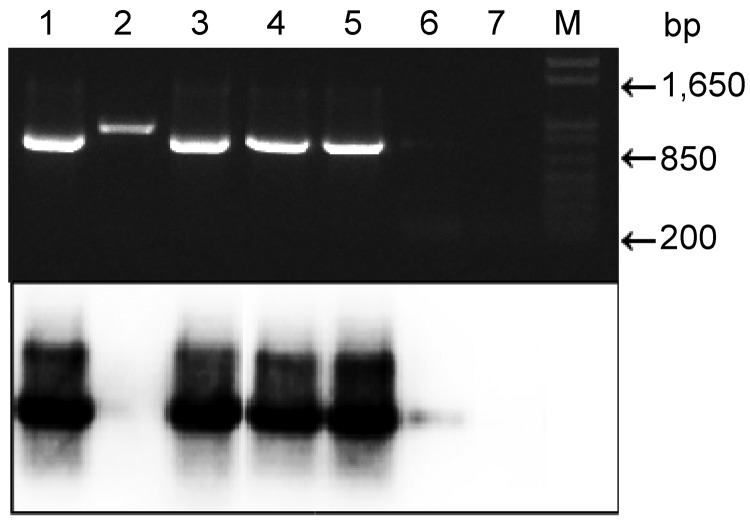
Top, agarose gel electrophoresis of nested PCR products of human fecal samples amplified with primers (pairs DW2-F + DW2-R and CYTB1-F + CYTB2-R) and stained with ethidium bromide. Bottom, autoradiograph of a Southern blot of the same gel. α-^32^P-ATP–labeled acytochrome B gene of *Plasmodium falciparum* was used as a probe. Lanes 1–6, samples from humans with malaria (the infection sample in lane 6 is weak); lane 2, spurious amplicon; lane 7, sample from an uninfected person; lane M, 1-kb molecular mass (Invitrogen, Carlsbad, CA, USA).

We analyzed fecal and blood samples obtained from persons in Uganda and determined their suitability for identification of *Plasmodium* spp. Parasites in various stages were detected in 13 blood smears (Table); all were identified as *P. falciparum*. PCR amplified a band of expected size from blood of 15 persons, and a weak band was consistently obtained from 6 samples. Amplification of *Plasmodium* *cyB* from feces of these patients was equally efficient; only 2 negative samples were identified (Table). A good correlation was observed between intensity of PCR products obtained from blood and feces, indicating that low parasitemia levels result in poor amplification regardless of the biological material used.

**Table:** Comparative analysis of diagnostic methods for *Plasmodium* spp. with different biologic material from humans, Africa*

Sample origin, no.	Blood smear parasitemia (%)	Blood PCR	Fecal PCR	Fecal Southern blot
Buikwe, Uganda				
T2767/11	+ (0.31)	+	+	+
T2768/11	+ (0.10)	+	+	+
T2770/11	+ (0.11)	+ (w)	+	+
T2774/11	+ (0.5)	+	+	+
T2775/11	+ (0.90)	+	+	+
T2776/11	+ (0.16)	+	+	+
T2777/11	+ (0.24)	+	+	+
T2779/11	+ (0.40)	+ (w)	+	+
T2781/11	+ (0.65)	+	+	+
T2785/11	+ (2.10)	+	+	+
T2786/11	+ (0.18)	+	+	+
T2772/11	+ (1.2)	+	–	–
T2778/11	+ (0.36)	–	+	+
T2778/11	–	+ (w)	+	+
T2782/11	–	+ (w)	+	+
T2780/11	–	+ (w)	–	+
Bayanga, Central African Republic	NA	+ (6)†	+ (6)†	+ (6)†

*+, positive; w, weak band; –, negative; NA, not available. †Samples from 6 persons.

PCR products for 2 fecal samples were then subjected to Southern blotting, which in 1 of 2 negative samples detected the cyB amplicon, which was not visible on an agarose gel (Figure). This method also confirmed that 1 band of slightly different mobility was a spurious amplicon (Figure). Therefore, with 1 exception, samples from 15 persons with *Plasmodium*–spp. positive blood samples contained *Plasmodium* DNA in their feces. Ten amplicons were sequenced and confirmed as being from *P. falciparum*.

We compared the capacity of blood and feces from persons clinically asymptomatic for malaria from the Central African Republic to amplify *cyB*. Blood and fecal samples from 5 of 28 persons showed bands of expected size, indicating asymptomatic or chronic malaria. Samples with positive and negative PCR results were also subjected to Southern blotting, which confirmed these results. In this dataset, the ability of blood and feces to accurately diagnose malaria was equal. All blood and fecal samples obtained from the 8 Europeans showed negative PCR results.

## Conclusions

We have shown that similar to apes, infected humans shed a detectable amount of *P. falciparum* in their feces, which correlates with results obtained by PCR. Southern blotting slightly enhanced the sensitivity of the PCR, but visual inspection of gel-resolved PCR products from feces was nearly equally sufficient (Table and Figure). We conclude that feces are as suitable as blood for malaria diagnostics for humans.

Although informative in terms of parasite diversity, prevalence of *Plasmodium* spp. amplified from feces of gorillas and chimpanzees was not determined in blood samples. A cautionary note regarding this issue and other issues has been reported (*14*). Our results show that in humans, *P. falciparum* efficiently penetrates the feces at levels detectable by PCR. Use of humans as proxies in our study validates previous estimates of malaria infection rates determined from feces of great apes (*3**,**4**,**7**,**9**,**12*). The diagnostic method we describe is suitable in situations in which feces are easier to obtain than blood and for use with small children.
